# ABE-VIEW: Android Interface for Wireless Data Acquisition and Control

**DOI:** 10.3390/s18082647

**Published:** 2018-08-13

**Authors:** Daniel M. Jenkins, Ryan Kurasaki

**Affiliations:** Molecular Biosciences and Bioengineering, University of Hawai’i at Mānoa, Honolulu, HI 96822, USA; rkurasak@hawaii.edu

**Keywords:** graphical user interface, open-source design, Arduino, rapid prototyping, virtual instrumentation, test equipment, Bluetooth, potentiostat

## Abstract

Advances in scientific knowledge are increasingly supported by a growing community of developers freely sharing new hardware and software tools. In this spirit we have developed a free Android app, ABE-VIEW, that provides a flexible graphical user interface (GUI) populated entirely from a remote instrument by ascii-coded instructions communicated wirelessly over Bluetooth. Options include an interactive chart for plotting data in real time, up to 16 data fields, and virtual controls including buttons, numerical controls with user-defined range and resolution, and radio buttons which the user can use to send coded instructions back to the instrument. Data can be recorded into comma delimited files interactively at the user’s discretion. Our original objective of the project was to make data acquisition and control for undergraduate engineering labs more modular and affordable, but we have also found that the tool is highly useful for rapidly testing novel sensor systems for iterative improvement. Here we document the operation of the app and syntax for communicating with it. We also illustrate its application in undergraduate engineering labs on dynamic systems modeling, as well as for identifying the source of harmonic distortion affecting electrochemical impedance measurements at certain frequencies in a novel wireless potentiostat.

## 1. Introduction

### 1.1. Overview/Objective

In recent years scientists and engineers have increasingly embraced the “open-source” and freeware movements to share software and hardware tools to help accelerate the pace of discovery [1]. Examples include software tools to analyze biological data such as from imagery [2], single cell RNA sequencing [3], and neurophysiology experiments [4]. Notably, more software tools are becoming available to directly interface with hardware for managing disparate sensor data over networks [5,6], automate the operation of laboratory instruments [7], and directly manage laboratory experiments [8]. Platforms such as Arduino and Raspberry Pi, with highly versatile open-source hardware, friendly development environments, and large communities of users have resulted in massive numbers of highly-customized sensor systems to address needs in precision agriculture [9,10], plant phenotyping [11], particle physics [12], geosciences [13,14], long-term low-power environmental monitoring [15], micro-scale process control [16], and other analytical [17,18,19] and educational [12,20,21,22] applications.

In this manuscript we demonstrate the features of a freely available Android app “ABE-VIEW” [23] intended to provide a versatile wireless graphical user interface (GUI) for customized hardware assemblies equipped with Bluetooth radio [20]. The primary advantage of ABE-VIEW is that available elements in the GUI have already been coded in XML (a user interface markup language) within the Android app itself, and are added/configured completely through simple ASCII-coded serial instructions from the remote device over a Bluetooth connection. This obviates the need for the user to become adept with separate software tools to develop source code, markup files, and/or to configure and manage html or other form data as would generally be required to develop a custom user interface. These advantages are most compelling for quickly developing and troubleshooting simple wireless data acquisition and control applications where user interaction and/or graphical data display is required. The app may also be used to quickly test and troubleshoot different components and functionalities of other more sophisticated systems that include Bluetooth hardware [24,25,26,27]. It is less suitable for applications where sophisticated data processing is required such as for machine vision, where reliable long-term operation is critical such as in commercially deployed industrial control systems and biomedical devices, or where more than one wireless system needs to communicate simultaneously.

In this manuscript we provide a general comparison of ABE-VIEW to similarly conceptualized software tools (Section 1.2), a basic user guide to the networking approach and syntax for communication (Section 2.2) with template source code (Supplemental materials), along with example applications of ABE-VIEW for data acquisition and control of simple systems in an undergraduate engineering lab (Section 2.3 and Section 3.2) and for testing/troubleshooting issues in a more sophisticated custom hardware design outfitted with Bluetooth (Section 2.4 and Section 3.3).

### 1.2. Background/Related Works

A variety of software tools are available to build rich graphical interfaces for custom instrumentation, but generally these require developers to be adept in more than one development environment and programming language. For example, Windows Forms built on the Microsoft.NET framework enables rich GUI features to respond to user interactions with an event-driven application, but requires familiarity with relatively sophisticated and proprietary integrated development environment (IDE; Microsoft Visual Studio, Microsoft Corp., Redmond, WA, USA) to develop a custom interface, and does not directly support software classes for interfacing to customized hardware. Some open-source analogs to Windows Forms are available, i.e., Koda Forms [28], but these still require that the GUI be developed in a separate software environment and are most suitable for user interaction with a custom software application within Windows rather than with customized hardware. ProviewR [29] is an open-source object-oriented development environment to network computers around process control of standard unit operations such as pumps and fans, using an architecture reminiscent of “ladder-logic” of traditional programmable logic controllers, but again requires separate configuration of an interface using a syntax that is potentially alien to beginning developers. While numerous tools have become available recently to facilitate the development of GUIs for controlling customized hardware, we believe that there are several compelling advantages of ABE-VIEW, notably again the approach that the GUI is configured directly from the remote device by ASCII coded serial instructions communicated wirelessly over Bluetooth. MIT app inventor [30,31,32] is a browser based graphical programming tool for composing a customized app/GUI, that can be configured to communicate through the Android Bluetooth, but the platform still requires that the custom interface be developed separately from the software operating on the remote device. Development of apps through MIT app inventor requires a “companion” app on the Android device, connected through internet to the browser-based developer tool. Blynk [33] is an app that can be installed directly on Android (or iOS), with a rich configurable interface with standard libraries to support commonly used open source hardware (i.e., Arduino and Raspberry Pi), but while the development interface is highly intuitive it still requires user configuration within the app in addition to the customized source code on the remote device. Blynk is operated through a proprietary server so it cannot be used as a direct interface to the test instrument without registering the app and connecting through the internet. Several options, in various states of development and documentation, are available for more direct local communication with a remote device. These include “Arduino Graph” [34], which requires snippets of custom code running on a personal computer (PC) to communicate with the software on the remote instrument, but the display is rather crude, not interactive, and generally restricted to a PC using a wired serial to USB interface. Somewhat more developed are “Instrumentino” [35] and “SerialComInstruments” [36] Windows-based free softwares for configuring graphical interfaces to interact with remote microcontroller systems through simple serial communication, with rich options for graphical display and control elements that send coded data back and forth to the microcontroller using a conserved syntax. However, these tools still require that the user configure the interface within the Windows app completely independently of (and in coordination with) the software running on the remote instrument. They are also restricted to operating on PCs through a wired USB to serial connection. In principle, ABE-VIEW is most similar to the “Arduino Dashboard” (a Windows-based executable project developed with Visual Basic) and “EzScrn” (a Python-based program with a browser interface, requiring installation of Python 2.7 interpreter) [37,38] in that elements in the GUI are enabled and configured by coded serial instructions from the remote device, and interaction with these elements results in coded instructions back to the remote device that are parsed to execute a desired function in the hardware (i.e., illuminate an LED). ABE-VIEW can be more convenient to operate in that it can be directly installed through Google Play onto a highly portable Android phone or tablet with no additional software dependencies, and connects directly to the remote instrument through Bluetooth. The ABE-VIEW interface also has some potentially improved features relative to previously published projects, for example the resolution of the controls is configurable, and data incoming to the app can easily be recorded into a standard comma delimited file for subsequent sharing and analysis. A systematic comparison of the features, strengths, and weaknesses of various software tools for facilitating GUI or custom instrumentation is summarized in Table 1.

The inspiration for ABE-VIEW was to facilitate a transition away from reliance on powerful yet expensive applications and hardware [22,39] for fundamental courses on instrumentation and control, in favor of increasingly capable open-source hardware options [40] and the compactness/ubiquity of smart-phones and tablets [18,41]. In the teaching context, development with ABE-VIEW allows students with basic coding skills to focus on experimental design, system modeling, and data analysis. A secondary motivation for developing ABE-VIEW was to facilitate the testing of new sensor technologies to help lower development costs and accelerate their transition into the marketplace of ideas. Correspondingly we illustrate example applications of the app in two design applications in an undergraduate engineering laboratory course on dynamic systems modeling (precision control of a customized positive displacement pump, and design/analysis of a heat exchanger in a thermal cycling application), as well as for identifying the source of harmonic distortion in a prototype wireless potentiostat currently under development that incrementally builds on previous open-source hardware designs [42,43]. Supplementary materials, including hardware schematics and Arduino source code templates, are shared to help facilitate adaptation of the app for new applications.

## 2. Materials and Methods

### 2.1. Overview

Details in the Materials and Methods below are provided primarily as documentation about the behavior of the app, and as a guide for new users to use the app to communicate with new customized hardware assemblies. Included is a comprehensive summary of GUI elements available, how they are explicitly populated on the interface from coded instructions from the connected device, and how to decode instructions/information sent from the app in response to user interaction with elements on the GUI. Supplemental materials include a hardware schematic and an associated template sketch from the Arduino IDE that includes code to set up every available GUI element, communicate data, concatenate data into dynamic ArrayLists, and decode instructions returning from user interactions on the GUI. We have also attempted to summarize how data communicated to the app is handled including options to save and share data in a comma delimited (.csv) file format. To illustrate use of the app in a pedagogical context in an undergraduate engineering lab, we have provided a brief description of two laboratory design assignments, with more detailed handouts as supplemental materials. To illustrate use of the app in an iterative hardware design/testing process, we have described the use of the app to control a customized wireless potentiostat system to identify the source of severe harmonic distortion in AC impedance measurements.

### 2.2. General Architecture of ABE-VIEW

**Figure 1:**
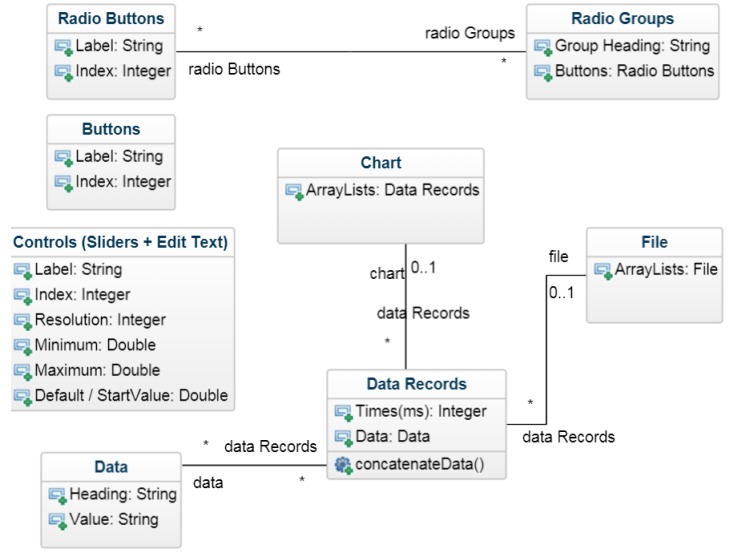
Class Diagram for ABE-VIEW app illustrating the attributes of various elements of the GUI, as well as the relationship between data communicated to the app and data records that are plotted on an optional chart or saved as a comma delimited file.

**Figure 2:**
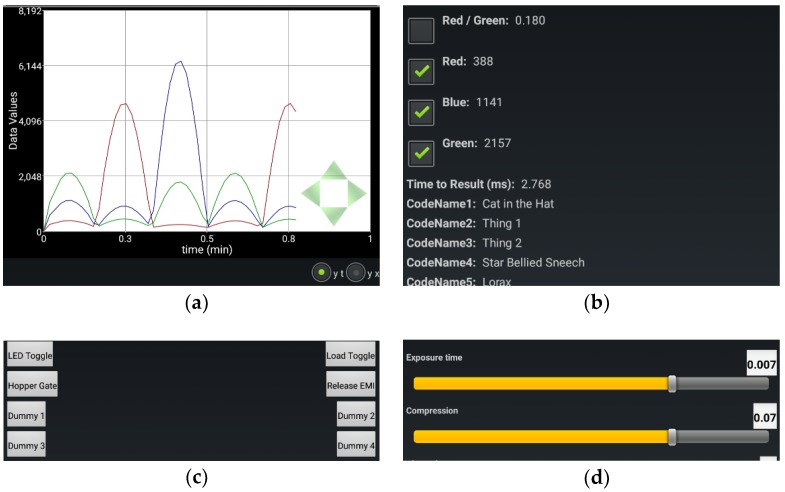

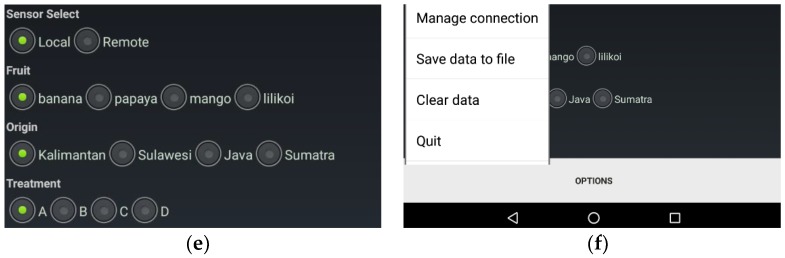
Illustration of ABE-VIEW graphical interface with customizable elements including: (**a**) interactive chart; (**b**) up to 16 data fields with text headings; (**c**) up to 8 buttons; (**d**) up to 8 numerical controls with customizable range and resolution; (**e**) up to 4 radio groups with up to 4 radio buttons each, and; (**f**) options menu while connected to remote instrument. All elements between the chart and options button are contained in a scrollview to ensure they are all accessible to the user.

All applications demonstrated in this work use ABE-VIEW [23], a freely available app with a flexible GUI (Figure 1) populated by serial ASCII-coded instructions communicated from the remote device over Bluetooth. In practice, data to and from the remote device is communicated serially through a universal synchronous/asynchronous receiver-transmitter (USART) or universal asynchronous receiver-transmitter (UART) port (i.e., Arduino “Serial” class), interfaced to a serial to Bluetooth converter (i.e., RN42, Roving Networks, Los Gatos, CA, USA; available packaged on an easily interfaced evaluation module as BlueSMiRF Silver, SparkFun, Niwot, CO, USA). When the app makes a Bluetooth connection to the remote device, ABE-VIEW sends a single character code (‘s’) to request that instructions are returned to enable any of the graphical elements that can compose the interface, including a chart that is able to plot numerical data, up to 16 data “indicators” with user defined headings, 8 virtual buttons, 8 numerical controls with user defined precision and range, and 4 radio groups with up to 4 radio buttons each (Figure 2).

**Figure 3:**
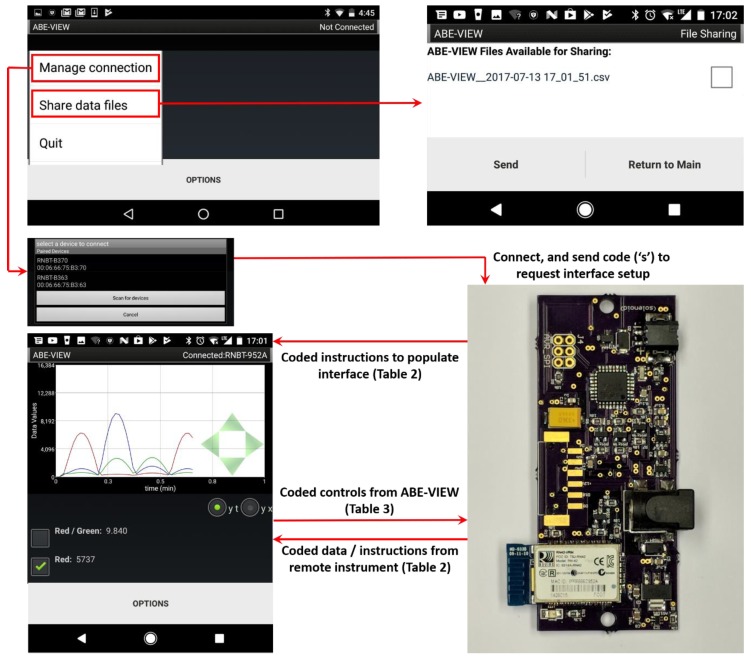
Flowchart for (Bluetooth) communications between ABE-VIEW and remote instrument/device. Upon making a Bluetooth connection with the remote device, ABE-VIEW sends a code ‘s’ that prompts the latter to return coded instructions to populate the interface. Thereafter, user interactions with the GUI result in coded instructions sent to the remote device, and coded data from the remote device are displayed by the app.

Options for each element in the GUI (i.e., headings, range, and resolution for numerical controls) are designated by tab delimited ascii text appended to the corresponding code (Table 2). Similarly, user interactions with elements in the GUI (button presses and numerical controls) result in standard coded text strings returned to the instrument that can be decoded accordingly (Table 3). A simplified flowchart for communication between devices is illustrated in Figure 3, and template Arduino source code is shared as Sketch S1 with a function to populate the app’s GUI, instructions to send data to the app, and functions to parse data returning from user interactions with buttons and numerical controls on the app.

Any element that is not specifically initialized following a Bluetooth connection does not appear in the GUI. Data “indicators” can have checkboxes associated with them (Table 1 code ‘h’) to select them to be plotted on (or removed from) the chart if the chart is active. Within the app, ArrayLists are assigned to each data element, and the most recent datum is concatenated into its corresponding ArrayList either at pre-defined intervals (Table 2 code ‘p’, typically issued once when connection is made), or on demand from the instrument by sending the code ‘u’ (Table 2). If the chart is active, it will plot all of the data in ArrayLists corresponding to checked data indicators. By default, the chart displays all selected data against time, but an alternative radio button “y-x” (Figure 2a) allows the user to plot data in ArrayLists against each other. If this option is selected, the first checked data group will be used for the “x” data, and all others will be used for “y”. The scales on the chart can be changed dynamically by swiping on the chart, and the origin can be moved by pressing any of the green arrows at the bottom right of the chart (Figure 2a; for “y-t” plots the origin of the time scale will never go below 0).

The options menu (Figure 2f) allows the user to clear the data in all ArrayLists (this will also clear the chart), or save all the ArrayLists with their corresponding headings into a comma delimited (.csv) file, with the first column including the times (in ms) which each row of data was added into their corresponding ArrayLists. An option allows these files to be shared by e-mail if the app is not actively connected to a device (Figure 3).

The illustrations provided in Figure 2 and Figure 3, and example strings in Table 2 and Table 3 come directly from an example using ABE-VIEW to interact with a custom circuit designed by undergraduate students to sort coffee cherries by color (Design S1). This device was designed to mount onto a chute, and it uses a digital color sensor (APDS-9960, Avago Technologies, San Jose, CA, USA) to measure the chromaticity of light reflecting off of cherries illuminated by diffused high power white LEDs. For wireless communication, the device has an integral Bluetooth radio (Roving Networks RN42) interfaced to the UART port of the microcontroller (ATMEGA328P, Atmel Corporation, Chandler, AZ, USA), which can be programmed through an Arduino device in the Arduino IDE using the built-in example sketch “ArduinoISP”. The device also can switch a load (up to 30V/3.6 A) through an n-channel metal-oxide-semiconductor field effect transistor (MOSFET), a feature originally intended to engage a pneumatic solenoid valve to actuate binary sorting. The waveforms displayed on the charts in Figure 2a and Figure 3 were recorded by alternately illuminating the color sensor with red, green and blue LEDs, adjusting intensities with a rectified sinusoidal current. The source code running on the device to control the GUI of ABE-VIEW, written in the Arduino IDE, is shared as Sketch S1, and may be used as a template to facilitate the use of ABE-VIEW for other applications.

### 2.3. Application of ABE-VIEW for Student Data Acquisition and Control

We have incorporated ABE-VIEW into engineering design courses. To illustrate example uses of the app, we have shared lab handouts from an undergraduate course in dynamic systems modeling, where electronics are assembled directly from commercially available open-source microcontrollers and sensor/controller evaluation boards. In one lab, students write firmware to enable ABE-VIEW to control a custom fabricated syringe pump operated by a stepper motor turning a lag screw to move the plunger (File S1). In a second lab/design experience, students are asked to design a heat sink to be used with a Peltier cooler, and analyze the performance of the system for thermal cycling (File S2).

### 2.4. ABE-VIEW for Rapid Device/Sensor Development

**Figure 4:**
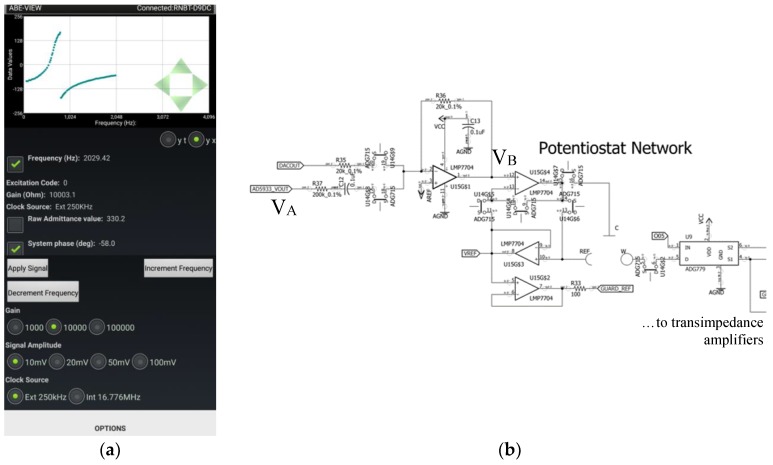
(**a**) Implementation of ABE-VIEW to set the frequency and amplitude of (**b**) the control signal to custom potentiostat network. The control voltage (V_B_) into the potentiostat network is composed of a weighted sum and inversion of the voltage from a digital synthesizer (V_A_) of a network analyzer chip (Analog Devices AD5933) with AC coupling, and a low pass filtered digital to analog converter voltage to apply arbitrary DC bias. This enables the device to perform any basic potentiometric or amperometric method in 2 or 3 electrode configuration, as well as electrochemical impedance analysis with arbitrary bias.

To illustrate the use of ABE-VIEW for testing and troubleshooting a custom hardware design, we used it to enable a user to interactively set the parameters of a control signal (Figure 4a) to a custom potentiostat network (Figure 4b). An integral Bluetooth (Roving Networks RN42) on the design (Design S2) allowed the firmware to be quickly adapted to operate with ABE-VIEW (Figure 4a; Sketch S2), with no additional hardware or software dependencies. The microcontroller used in the design (ESP8266 packaged on module ESP-12S, Espressif Systems, Shanghai, China) can be programmed directly through the Arduino IDE by importing the associated system dependencies, so the example sketch (S2) is also provided as using the Arduino file extension. This convenience facilitated the identification of sources of harmonic distortion in the control signal resulting in very large errors in the predicted electrochemical impedance spectra at certain frequencies, which enabled us to quickly make iterative design improvements to correct the issue.

The custom potentiostat (Design S2) is largely inspired by the component selection and design configuration of the high-performance open-source design reported previously by Dryden and Wheeler [42]. Our design has attempted to condense the footprint slightly, and include Bluetooth (integral Roving Networks RN42) and WiFi (ESP8266, Espressif Systems) capability to facilitate portability and/or to allow the system to be directly networked. Notably, our design has added a chip level network analyzer (AD5933, Analog Devices, Norwood, MA, USA) to enable the device to conduct Electrochemical Impedance Spectroscopy (EIS). The excitation signal from the AD5933 is provided by a digital synthesizer that can be operated at any of 4 voltage amplitudes, at a frequency that is set by controlling the incremental value added to a phase accumulator at fixed intervals with respect to the system clock [44]. In our design, the control signal to the potentiostat network (V_B_, Figure 4b) is set by summing a scaled (and AC coupled) version of the synthesizer signal (V_A_, Figure 4b) with the signal from a separate Digital to Analog Converter (DAC; AD5061, Analog Devices). The superposition of these signals allows EIS to be conducted at an arbitrary frequency and DC bias, with any of 4 pre-defined amplitudes. The AD5933 reports a complex representation of the system admittance based on the Discrete Fourier Transform (DFT) of 1024 current values sampled from an internal transimpedance amplifier with an externally configured gain, at a fixed frequency with respect to the system clock [44]. At low frequencies, significant errors can occur by not sampling a whole integer number of waveforms at the given frequency, so that the effective range of frequencies that can be analyzed by the AD5933 operating with the internal 16.776 MHz clock is about 1 kHz to 100 kHz (with the upper end of the range limited by low pass filtering of the synthesizer signal). We have accurately evaluated EIS spectra between 0.1 Hz up to about 70 Hz by generating the desired waveform directly with the DAC, and evaluating the complex value of the corresponding element from the DFT of working current values sampled from a separate transimpedance amplifier. To fill in the gap in the EIS spectra we included a 250 kHz external clock that can be selected to provide an alternative sampling frequency and update interval to the AD5933 phase accumulator, in theory allowing admittances to be evaluated accurately between 15.29 Hz and 7812.5 Hz.

In the prototype design described above, we observed very serious systematic errors in the predicted admittances for signals around 750 Hz when operating the AD5933 with the 250 kHz external clock. To determine the source of these errors, we quickly adapted the device firmware to operate with ABE-VIEW to provide an easy way to control and maintain the characteristics of a desired signal into the potentiostat network (Figure 4a; Sketch S2). The circuit was then probed with a digital storage oscilloscope (TDS2012B, Tektronix Inc., Beaverton, OR, USA) to compare the synthesizer signal to other signals in the network to identify any sources of distortion that could potentially be corrected in a revised design. For these tests we configured the network to operate in a 2-electrode configuration, applied no DC bias, and used passive resistors for the network load. The ABE-VIEW interface was configured to also enable the control of the transimpedance amplifier gain from the network, to help determine if the electrode network and downstream measurement electronics had an effect on distortion of the control signal.

## 3. Results

### 3.1. Overview

We share below results using the ABE-VIEW app in a pedagogical context with customized hardware assemblies wired together quickly from commercially available evaluation modules, and screenshots illustrating customized GUIs and graphical data from these systems using customized Arduino code that students can quickly adapt from scratch or from a template (i.e., Sketch S1). Since we believe that the app can also be a very useful tool for iterative hardware design and testing, we have also included a somewhat more detailed description of results using ABE-VIEW with a customized wireless potentiostat device to identify the source of harmonic distortion in AC impedance measurements.

### 3.2. ABE-VIEW for Flexible Data-Acquisition and Control

**Figure 5:**
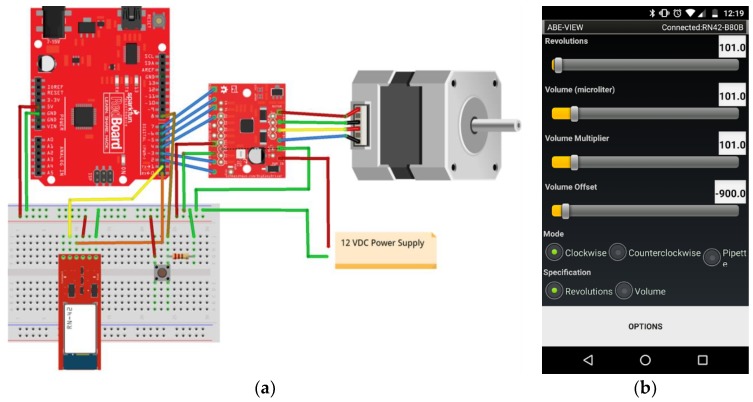
(**a**) Motion control system assembled by students using off-the-shelf components (Sparkfun RedBoard, BlueSMiRF Silver, Big Easy Motor Driver, and 12 V bipolar 200 steps/rev stepper motor), and (**b**) student populated ABE-VIEW interface to control the system to operate the motor to drive a custom pipette/syringe pump.

**Figure 6:**
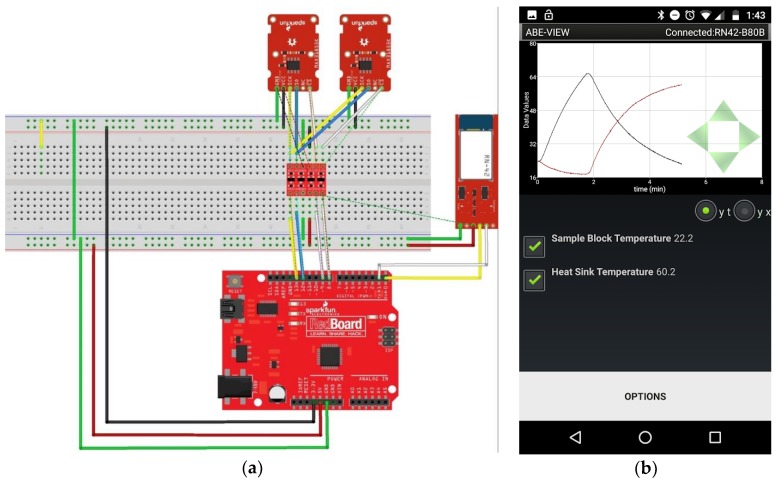
(**a**) Data acquisition system assembled by students using off the shelf components (Sparkfun RedBoard, BlueSMiRF Silver, 2 MAX31855 thermocouple breakout boards with type K thermocouple, and bi-directional logic level converter) to record the dynamic performance of a thermal cycler with a student designed heat sink, and; (**b**) student populated ABE-VIEW interface for data acquisition.

Junior level undergraduate students were able to assemble simple electronic systems by wiring together Arduino-compatible microcontroller boards and other off-the-shelf evaluation boards. To communicate with ABE-VIEW, students used a Bluetooth module (BlueSMiRF Silver) that could be powered from the regulated 5V from Arduino and connected directly to the serial UART port, and which includes built-in logic level shifting to work with the 3.3 V logic level Bluetooth radio on board. Students were able to quickly and independently write sketches using Arduino IDE to populate the ABE-VIEW interface to intuitively control and record data from these systems (Figure 5 and Figure 6).

### 3.3. ABE-VIEW for Custom Device Testing and Troubleshooting

**Figure 7:**
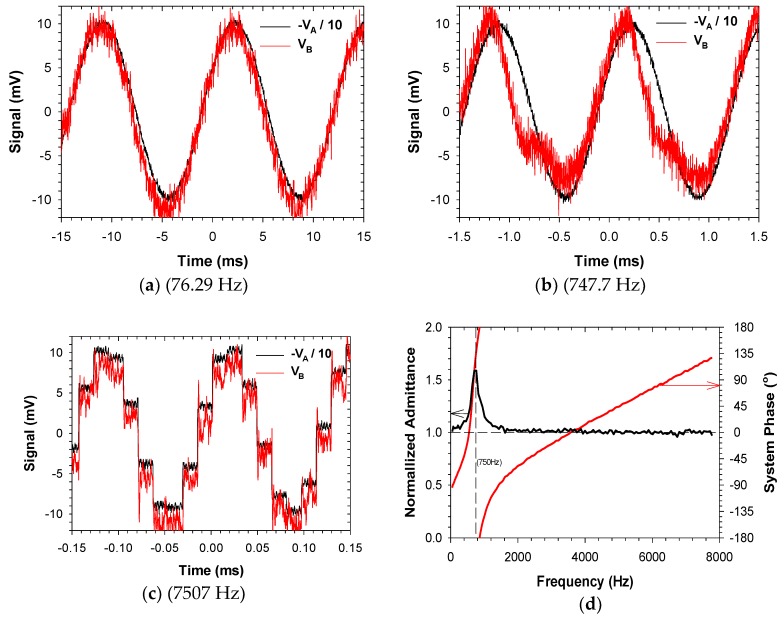
Recorded signals from digital synthesizer of network analyzer (V_A_) and output of summing junction (V_B_) for potentiostat operating to deliver 10 mV_peak_ at (**a**) 76.29 Hz; (**b**) 747.7 Hz, and; (**c**) 7507 Hz, with network analyzer running on 250 kHz external clock. The control signal V_B_ consistently exhibits a few more mV of random noise, but large additional harmonic distortions near 750 Hz result in HH (**d**) very significant errors in admittance and system phase of a resistor as predicted by the network analyzer. Note that the synthesizer output V_A_ is inverted and divided by 10 to scale with the predicted value of control signal V_B_, and the frequency at which the phase accumulator is updated for the synthesizer (62.5 kHz) is easily observable in the 7507 Hz signal (**c**).

By adapting the custom potentiostat firmware to work with ABE-VIEW to easily set the control signal into the potentiostat network, we were able to quickly identify the origin of harmonic distortion affecting EIS measurements near 750 Hz. Interestingly, distortions in system phase and admittance magnitudes (normalized to the predicted values for the given load resistor) were highly conserved for given control signals, independent of load resistor and transimpedance amplifier gain. Observed distortions of signal V_B_ relative to V_A_ were reproduced with high fidelity at every location in the reference/counter electrode control circuit, and indeed were independent of whether the DAC signal was removed, and whether V_B_ was disconnected from the network control amplifiers (data not shown). Distortion was most pronounced near 750 Hz, resulting in the extremely large errors in admittance and system phase reported by AD5933 (Figure 7), evidently originating in the summing amplifier used to construct the control signal to the network. The distortion at this frequency results in a sharp peak in the predicted admittance value for the 10 mV_peak_ amplitude signal, and correspondingly an inflection point in the system phase vs. frequency (Figure 7d). While a significant change is expected in the observed “system phase” with frequency (which must be calibrated for when using the device to predict the phase of actual network impedance), the relationship is typically highly linear in the absence of control signal distortion [44]. Interestingly, at all tested signal amplitudes the observed distortion (as estimated by the reported complex admittance) was numerically consistent with a resonant signal with peak amplitude of about 25 mV, and approximately 180 degrees out of phase with the control signal, at 750 Hz (analysis not shown). This “destructive” interference overwhelmed the smallest desired control signal (10 mV) and resulted in larger predicted admittance (i.e., Figure 7d), and very dramatic changes in system phase as the superposition of the control signal and harmonic rapidly rotated around the phase plane at frequencies near the harmonic. Control signals with larger putative magnitudes (20 mV, 50 mV, and 100 mV) resulted in underestimated admittance values as superposition with the harmonic signal resulted in signals smaller in magnitude than the desired control signals (Figure S1a). At the two highest values of the putative control signal amplitudes (50 mV and 100 mV), the harmonic signal was never larger than the control signal, so that non-linearities in system phase were moderated (Figure S1b). Closer investigation into the source of the summing amplifier distortion showed that it was due to instability in the analog virtual ground due to overloading the precision analog voltage reference with the network analyzer.

From initial evaluation of the potentiostat performance, we were inclined to correct for harmonic distortions in software based on their reproducible behavior, and independence from the network load and transimpedance amplifier gain. However, given the non-linear (i.e., less predictable) behavior of more complex electrochemical networks we chose instead to investigate the source of the distortion to correct it in hardware. As a result, we redesigned the coupling between the AD5933 synthesizer and potentiostat network, and modified the power regulation for the network analyzer to eliminate the observed distortions. These modifications successfully eliminated the distortion, and we have successfully used the revised prototype to make accurate EIS measurements across the spectrum from 0.1 Hz to 100 kHz.

## 4. Discussion

Our primary objective of this manuscript is to facilitate the adaptation of ABE-VIEW by new users for unique applications, so we have tried to concisely document the features, program flow, and syntax for communicating with it. The approach of the app is somewhat unique among standalone applications in that the flexible GUI is configured entirely from instructions from the remote instrument so that users are only required to develop firmware for the remote device without any additional coding required for the Android device. As communication with the app is based on simple ascii coded instructions, the remote firmware can easily be developed using virtually any development environment (such as the user-friendly Arduino IDE), and the physical link can easily be achieved with simple serial to Bluetooth radios. This allows novel hardware configurations to be rapidly assembled and tested without building in additional displays, interfacing hardware, proprietary cables, or relying on desktop applications for an interface. Our initial motivation was to use this as a versatile and modular platform to support instruction in engineering courses at the University of Hawaii. In this context the effort has been quite successful, as students can easily configure the interface and focus on mastery of a single IDE, understanding more fundamental principles of instrumentation, and other analytical skills related to complex system dynamics. The app has also been very useful to us in testing and troubleshooting novel instrumentation systems, as illustrated in our identification of the origin of harmonic distortion in a wireless potentiostat system. To encourage new users to use ABE-VIEW, we have provided supplemental materials including template source code (Sketch S1) with related hardware designs that can be used to rapidly adapt the app for new applications. It is our sincere hope that this work will be useful to others in accelerating the development of novel instruments and applications that we can, in turn, benefit from.

## Figures and Tables

**Table 1:** Comparison of ABE-VIEW features to alternative software for GUI development for custom hardware. The key features that we believe facilitate use by inexperienced developers are flagged in red.

Tool	Standalone Mobile App	No Additional Software Dependencies	Android	iOS	PC/Windows/Linux	Direct Support for Wireless Bluetooth	GUI Fully Configured by Remote Instrument	Easy user Options to Save/Share Data	High Quality Graphical Displays	Numerical Controls with Arbitrary Precision	Easily Scalable Structured Coding (Cut/Paste/Edit)	Can Operate without Internet	Can Connect to Hardware through Internet
ABE-VIEW	√	√	√	×	×	√	√	√	√	√	√	√	×
Blynk [33]	√	√	√	√	×	√	×	√	√	√	×	×	√
MIT App Inventor [30,31]	×	× ^1^	√	×	√ ^1^	√	×	√	√	√	×	√ ^1^	√
Arduino Dashboard [37]	×	×	×	×	√	×	√	√ ^2^	√	×	√	√	×
EzScrn [38]	×	×	×	×	√	×	√	×	×	×	√	√	×
Instrumentino [35]	×	×	×	×	√	×	×	√	√	√	√	√	×
SerialComInstruments [36]	×	×	×	×	√	×	×	√	√	√	×	√	×
Arduino Graph [34]	×	×	×	×	√	×	×	×	×	×	√	√	×
ProviewR [29]	×	×	×	×	√	×	×	×	√	√	√ ^3^	√	√

^1^ App/GUI is configured in a browser, with internet connection to a companion app. ^2^ All serial data is recorded in a logging window. ^3^ Development options include graphical editor or structured code (C, C++, Java, FORTRAN).

**Table 2:** Coded instructions from remote device to populate ABE-VIEW interface, and share data.

Code	Description	Options ^1^	Example Text String(s)	GUI Result
g	display chart	enable (0 or 1)	“g1\t”	Graph visible (i.e., Figure 2a)
h	configure data heading/options	heading index (0–15) enable checkbox for chart (0 or 1) heading text	“h2\t1\tBlue: \t”	Data (index 2) enabled with checkbox to plot on graph, with heading “Blue: (i.e., Figure 2b)
b	configure button	button index (0–7) button text/label	“b0\tLED Toggle\t”	Button (index 0) enabled, with label “LED Toggle” (i.e., Figure 2c)
c	configure numerical control	control index (0–7) resolution (integer–decimal digits) minimum value default/start value maximum value heading text	“c4\t3\t-0.015\t0.007\t0.015\tExposure time\t”	Numerical control (index 4) enabled, with 3 decimal place precision, range −0.015 to 0.015, default value 0.007, labelled “Exposure time” (Figure 2d)
r	configure radio buttons	radio group index (0–3) radio group text radio button 1 text radio button 2 text radio button 3 text radio button 4 text	“r0\tSensor Select\tLocal\tRemote\t\t\t” “r1\tFruit\tbanana\tpapaya\tmango\tlilikoi\t”	Radio group (index 0) enabled, group label “Sensor Select” with buttons “Local” and “Remote” Radio group (index 1) enabled, group label “Fruit”, with buttons “banana”, “papaya”, “mango”, “lilikoi” (i.e., Figure 2e)
d	send data	data index (0–15) data value	“d2\t1141\t”	New data element (“1141”) for data array index 2 (Figure 2b)
p	set period for automatically concatenating latest data into array(s)	period (in seconds)	“p10\t”	Most recently communicated value in each data field (and system time) will be automatically concatenated into its corresponding ArrayList every 10 s
u	concatenate most recent data into array(s)	n/a	“u”	Most recently communicated value in each data field (and system time) will immediately be concatenated into its corresponding ArrayList

^1^ Tab (“\t”) delimited configuration options appended to code.

**Table 3:** Coded information from ABE-VIEW to remote device indicating user interactions with GUI.

Code	Description	Options ^1^	Example Text String (s)	Response to User Action
s	device connected—request GUI configuration	n/a	“s”	User/app just connected to remote Bluetooth (request GUI configuration)
b	button pressed	button index (0–7)	“b0\t”	Button (index 0) pressed
c	control value changed	control index (0–7) control value	“c0\t100.0\t”	Control (index 0) set to 100.0
r	radio button pressed	radio button identifier (“ij”) where i is radio group (1–4) and j is button (1–4)	“r12\t”	Second button in first radio group was pressed

^1^ Tab (“\t”) delimited configuration options appended to code.

## Supplementary Materials

The following are available online at http://www.mdpi.com/1424-8220/18/8/2647/s1, Design S1: Coffee Cherry Color Sorter, Sketch S1: Coffee Cherry Color Sorter ABE-VIEW Example Template, File S1: Lab Handout for Precision Syringe Pump Control, File S2: Lab Handout for Heat Exchanger/Thermal Cycler Design and Testing, Design S2: ABE-Stat1_0_01 Potentiostat, Sketch S2: ABE-VIEW signal control for ABE-Stat, Figure S1: Normalized admittance and system phase for different signal amplitudes.

## References

[1] Dryden M.D.M., Fobel R., Fobel C., Wheeler A.R. (2017). Upon the Shoulders of Giants: Open-Source Hardware and Software in Analytical Chemistry. Anal. Chem..

[2] Rueden C.T., Schindelin J., Hiner M.C., DeZonia B.E., Walter A.E., Arena E.T., Eliceiri K.W. (2017). ImageJ2: ImageJ for the next generation of scientific image data. BMC Bioinform..

[3] Zhu X., Wolfgruber T.K., Tasato A., Arisdakessian C., Garmire D.G., Garmire L.X. (2017). Granatum: A graphical single-cell RNA-Seq analysis pipeline for genomics scientists. Genome Med..

[4] Zhang B., Dai J., Zhang T. (2017). NeoAnalysis: A Python-based toolbox for quick electrophysiological data processing and analysis. Biomed. Eng. Online.

[5] Mejías A., Herrera R., Márquez M., Calderón A., González I., Andújar J. (2017). Easy Handling of Sensors and Actuators over TCP/IP Networks by Open Source Hardware/Software. Sensors.

[6] Martínez E., Toma D., Jirka S., del Río J. (2017). Middleware for Plug and Play Integration of Heterogeneous Sensor Resources into the Sensor Web. Sensors.

[7] Lütjohann D.S., Jung N., Bräse S. (2015). Open source life science automation: Design of experiments and data acquisition via “dial-a-device”. Chemom. Intell. Lab. Syst..

[8] Chen X., Li H. (2017). ArControl: An Arduino-Based Comprehensive Behavioral Platform with Real-Time Performance. Front. Behav. Neurosci..

[9] Gao Y., Ramirez B.C., Hoff S.J. (2016). Omnidirectional thermal anemometer for low airspeed and multi-point measurement applications. Comput. Electron. Agric..

[10] Barnard H.R., Findley M.C., Csavina J. (2014). PARduino: A simple and inexpensive device for logging photosynthetically active radiation. Tree Physiol..

[11] Tovar J.C., Hoyer J.S., Lin A., Tielking A., Callen S.T., Elizabeth Castillo S., Miller M., Tessman M., Fahlgren N., Carrington J.C. (2018). Raspberry Pi-powered imaging for plant phenotyping. Appl. Plant Sci..

[12] Axani S.N., Frankiewicz K., Conrad J.M. (2018). The CosmicWatch Desktop Muon Detector: A self-contained, pocket sized particle detector. J. Instrum..

[13] Soler-Llorens J.L., Galiana-Merino J.J., Giner-Caturla J., Jauregui-Eslava P., Rosa-Cintas S., Rosa-Herranz J. (2016). Development and programming of Geophonino: A low cost Arduino-based seismic recorder for vertical geophones. Comput. Geosci..

[14] Soler-Llorens J.L., Galiana-Merino J.J., Giner-Caturla J.J., Jauregui-Eslava P., Rosa-Cintas S., Rosa-Herranz J., Nassim Benabdeloued B.Y. (2018). Design and test of Geophonino-3D: A low-cost three-component seismic noise recorder for the application of the H/V method. Sens. Actuators A Phys..

[15] Beddows P.A., Mallon E.K. (2018). Cave Pearl Data Logger: A Flexible Arduino-Based Logging Platform for Long-Term Monitoring in Harsh Environments. Sensors.

[16] Husain A.R., Hadad Y., Zainal Alam M.N.H. (2016). Development of Low-Cost Microcontroller-Based Interface for Data Acquisition and Control of Microbioreactor Operation. J. Lab. Autom..

[17] De Morais C.D.L.M., Carvalho J.C., Sant’Anna C., Eugênio M., Gasparotto L.H.S., Lima K.M.G. (2015). A low-cost microcontrolled photometer with one color recognition sensor for selective detection of Pb^2+^ using gold nanoparticles. Anal. Methods.

[18] Shen H.-Y., Chen Y.-C., Hsu C.-H. (2017). A Power Frequency Sensing Device Using an Arduino Device and Zero-Crossing Algorithm and Its Implementation on Android App. Sensors Mater..

[19] Segura F., Bartolucci V., Andújar J. (2017). Hardware/Software Data Acquisition System for Real Time Cell Temperature Monitoring in Air-Cooled Polymer Electrolyte Fuel Cells. Sensors.

[20] Jin H., Qin Y., Pan S., Alam A.U., Dong S., Ghosh R., Deen M.J. (2018). Open-Source Low-Cost Wireless Potentiometric Instrument for pH Determination Experiments. J. Chem. Educ..

[21] Grinias J.P., Whitfield J.T., Guetschow E.D., Kennedy R.T. (2016). An Inexpensive, Open-Source USB Arduino Data Acquisition Device for Chemical Instrumentation. J. Chem. Educ..

[22] Nichols D. (2017). Arduino-Based Data Acquisition into Excel, LabVIEW, and MATLAB. Phys. Teach..

[23] Jenkins D.M. Google Play, ABE-VIEW. Https://play.google.com/store/apps/details?id=com.uhmbe.DAQCTRL&hl=en_US.

[24] Brunelli D., Farella E., Giovanelli D., Milosevic B., Minakov I. (2016). Design Considerations for Wireless Acquisition of Multichannel sEMG Signals in Prosthetic Hand Control. IEEE Sens. J..

[25] Rossi M., Khouia A.O., Lorenzelli L., Brunelli D. Energy neutral 32-channels embedded readout system for IoT-ready fitness equipments. Proceedings of the 2016 IEEE Sensors Applications Symposium (SAS).

[26] Brunelli D., Tadesse A.M., Vodermayer B., Nowak M., Castellini C. Low-cost wearable multichannel surface EMG acquisition for prosthetic hand control. Proceedings of the 2015 6th IEEE International Workshop on Advances in Sensors and Interfaces, IWASI 2015.

[27] Brunelli D., Farella E., Rocchi L., Dozza M., Chiari L., Benini L. Bio-feedback system for rehabilitation based on a wireless body area network. Proceedings of the 4th Annual IEEE International Conference on Pervasive Computing and Communications (PerCOM 2006)—Workshop UbiCare.

[28] Koda Forms. http://koda.darkhost.ru/page.php?id=index.

[29] ProviewR. http://www.proview.se/v3/.

[30] MIT App Inventor. http://appinventor.mit.edu/explore/index-2.html.

[31] MIT App Inventor 2. http://ai2.appinventor.mit.edu/Ya_tos_form.html.

[32] Mnati M., Van den Bossche A., Chisab R. (2017). A Smart Voltage and Current Monitoring System for Three Phase Inverters Using an Android Smartphone Application. Sensors.

[33] Blynk. https://www.blynk.cc/.

[34] Arduino Graph. https://www.arduino.cc/en/tutorial/Graph.

[35] Koenka I.J., Sáiz J., Hauser P.C. (2015). Instrumentino: An Open-Source Software for Scientific Instruments. Chim. Int. J. Chem..

[36] SerialComInstruments. http://www.serialcominstruments.com/instrument.php.

[37] Arduino Dashboard. http://www.mathias-wilhelm.de/arduino/projects/arduino-dashboard/.

[38] EzScrn. https://forum.arduino.cc/index.php?topic=312547.0.

[39] Sandesh R.S., Venkatesan N. (2016). LabVIEW-based design and control of five-digit anthropomorphic robotic hand using EEG signals. Int. J. Biomed. Eng. Technol..

[40] Cvjetkovic V.M., Matijevic M. (2016). Overview of Architectures with Arduino Boards as Building Blocks for Data Acquisition and Control Systems. Int. J. Online Eng..

[41] Priye A., Wong S., Bi Y., Carpio M., Chang J., Coen M., Cope D., Harris J., Johnson J., Keller A. (2016). Lab-on-a-Drone: Toward Pinpoint Deployment of Smartphone-Enabled Nucleic Acid-Based Diagnostics for Mobile Health Care. Anal. Chem..

[42] Dryden M.D.M., Wheeler A.R. (2015). DStat: A Versatile, Open-Source Potentiostat for Electroanalysis and Integration. PLoS ONE.

[43] Rowe A.A., Bonham A.J., White R.J., Zimmer M.P., Yadgar R.J., Hobza T.M., Honea J.W., Ben-Yaacov I., Plaxco K.W. (2011). CheapStat: An Open-Source, “Do-It-Yourself” Potentiostat for Analytical and Educational Applications. PLoS ONE.

[44] Analog_Devices AD5933 Datasheet, Rev F. http://www.analog.com/media/en/technical-documentation/data-sheets/AD5933.pdf.

