# Supplementary material for: ABE-VIEW: Android Interface for Wireless Data Acquisition and Control
Source: Sensors (Basel). 2018 Aug 13;18(8):2647. doi: 10.3390/s18082647 (PMC6111993; doi:10.3390/s18082647)
Supplement: Supplementary file 1 [file sensors-18-02647-s001.zip › Figure S1.docx]

|   (**a**) |   (**b**) |
| --- | --- |

**Figure S1.** Values of (**a**) normalized admittance magnitude and (**b**) system phase predicted by AD5933 as a function of frequency, for different putative values of applied control signal V_B_. Severe harmonic distortion at 750 Hz, numerically equivalent to about a 25 mV_peak_ signal approximately 180° out of phase with the control signal, results in destructive interference such that putatively small applied control signals are susceptible to greater magnitudes of errors, and extreme swings in system phase where the harmonic signal exceeds the desired control signal in magnitude. Predicted admittance magnitudes (normalized to expected values from load resistor) and system phase were observed to depend on frequency and amplitude of the desired control signal, but were independent of the value of the load resistor and gain of the transimpedance amplifier.
