# Supplementary material for: ABE-VIEW: Android Interface for Wireless Data Acquisition and Control
Source: Sensors (Basel). 2018 Aug 13;18(8):2647. doi: 10.3390/s18082647 (PMC6111993; doi:10.3390/s18082647)
Supplement: Supplementary file 1 [file sensors-18-02647-s001.zip › File S1.docx]

BE 350L

# Lab 3A Handout - Stepper Motor Control

# AEI 123 October 19, 2017

**AEI 123 October 26, 2017**

**Report Due November 9, 2017**

**Revision Due November 23, 2017**

In this lab exercise, you will use your Arduino skills to develop the code to control the speed of a stepper motor. Your code will issue commands to a stepper motor controller circuit “Big Stepper Driver”, via controlled pulses from a digital I/O port on the Redboard. The controller will interpret those timed pulses and sequence current to the individual motor windings accordingly. Stepper motors (a class of “brushless DC” motors that are commutated electronically by energizing individual coils in the stator, in contrast to traditional DC motors which alternate the current through brush contacts as the rotor turns) are commonly used in motion control applications because the position can be easily controlled down to the resolution of a single (or even a half) “step” without the use of sensory feedback. The motor you will use in lab (Mercury motor - spec sheet attached) is a 12V (nominal) two-phase bipolar stepper motor with 200 steps per revolution of the rotor.

You will be provided a sketch with the code for the user interface (menu) that prompts the user to select for the following options.

1. Move forward a fixed number of steps upon button press.
2. Move backward a fixed number of steps upon button press.
3. Pipette mode. Uptake media upon first button press. Dispense upon second button press.

For the first part of the lab exercise, we will create an algorithm for a controlled acceleration to the specified speed, continue at the specified speed until within 50 steps of the total steps, and then slow the speed to zero when the total number of steps have been traveled. The mechanism for turning the motor by a single step is to create a square wave pulse by turning a digital port on and then off. The motor speed will be dependent on the frequency of the pulses, so part of your algorithm will be to determine the appropriate delay between each pulse.

For example, a speed of 500 steps/sec is accomplished by pulsing the digital port HIGH for 1 ms then low for 1 ms for a total of 2 ms per step. This is the close to the maximum speed with this hardware configuration (0.7 ms delay). Similarly a speed of 1 steps/sec is accomplished by pulsing the digital port HIGH for 500 ms then low for 500 ms for a total of 1000 ms per step.

1. *Open the provided stepper motor sketch “Steps test”. An external library will not be required for this application.*
2. *Write the code for the Steps function. The Steps function should move the motor through the specific number of steps from the revolutions setting. This function will calculate the required delay and turn the digital output port HIGH and then LOW for each step required for motion.*
3. *Test your code by observing the motor rotate in both directions through several angles of shaft rotation.*
4. *Open the provided stepper motor sketch “Lab 3 Start”. Insert the Steps function into the main loop to implement the menu functions.*
   1. *Move counter clockwise a fixed number of steps upon button press.*
      1. *Set direction.*
      2. *Implement Steps function.*
      3. *Reset ACTIVATE status.*
   2. *Move backward a fixed number of steps upon button press.*
      1. *Set direction.*
      2. *Implement Steps function.*
      3. *Reset ACTIVATE status*
   3. *Pipette mode (part 1). Sample upon first button press.*
      1. *Set direction.*
      2. *Implement Steps function.*
      3. *Reset ACTIVATE status*
   4. *Pipette mode (part 2). Dispense upon second button press.*
      1. *Set direction.*
      2. *Implement Steps function.*
      3. *Reset ACTIVATE status*
5. *Upload the sketch to the Redboard.*
6. *Power down the Redboard.*
7. *Connect the Redboard to the stepper motor controller.*

| Redboard | Controller Input |
| --- | --- |
| 2  3  4  5  6  7  GND  VCC | STEP  DIR  MS1  MS2  MS3  EN  GND  5V |

1. *Make sure that the Stepper motor is connected correctly to the controller. The motor is a 12 VDC nominal bipolar stepper motor with 200 steps/ revolution. The motor leads are connected to the controller as below.*

| Motor | Controller Output |
| --- | --- |
| Red | A1 |
| Green | A2 |
| Yellow | B1 |
| Blue | B2 |

1. *Connect the drive power leads to an isolated power supply at 12 VDC (M+, GND). Do not turn on power supply.*
2. *Check all connections before turning devices on. Turn on Redboard first and then power supply.*
3. *Test your code with the 360° scale and a stop watch for forward and backward movement. Also make sure all menu functions work.*

Figure 1. Sample square wave signal to control the speed of a stepper motor with the “Big Easy Driver” controller.**BE 350L**

**Lab 3B Handout – Automated Pippetor**

For this lab you will be testing the ability of an automated pipetter to dispense liquid in both accurate and repeatable volumes. You will use the pipette driver program to run your motor controller subprogram.

1. The dispensing cycle is described in the table below. As before, the system should wait for your manual confirmation before taking a sample and dispensing a sample.

| **Action** | **Algorithm** |
| --- | --- |
| ***Manual Confirmation*** | |
| Take Sample | 1. Raise plunger from initial position to some top position. |
| ***Manual Confirmation*** | |
| Dispense Sample | 1. Lower plunger from top position to initial position |
|  | 1. Lower plunger from initial position to purge position. |
|  | 1. Raise plunger from purge position to initial position. |

1. Develop an experimental procedure to adequately test the ability of the pipette apparatus to dispense water. Use your experience from previous lab exercises to generate a series of tests where the travel of the plunger is compared to the volume of water dispensed and calibrate the device. Evaluate the calibration by performing additional tests while controlling the motor to dispense calibrated volumes rather than displacement settings.
2. Ensure the data set is adequate in both range and repetition to analyze the accuracy and repeatability of the pipette.
3. You will be able to measure the mass of water dispensed with an analytical balance.
4. **Prevent the motor from driving the plunger too deep and hitting the bottom of the syringe tube.
