# Supplementary material for: ABE-VIEW: Android Interface for Wireless Data Acquisition and Control
Source: Sensors (Basel). 2018 Aug 13;18(8):2647. doi: 10.3390/s18082647 (PMC6111993; doi:10.3390/s18082647)
Supplement: Supplementary file 1 [file sensors-18-02647-s001.zip › File S2.docx]

| BE 350L |  |  |
| --- | --- | --- |
| Final Design Project |  |  |
| Report Due December 7, 2017 |  |  |
|  |  |  |

**Design of thermal cycling unit for DNA amplification through polymerase chain reaction (PCR).**

This lab exercise will involve the design of hardware and software components of a temperature control system. The intended application is a thermal cycler to enable the different steps required for polymerase chain reaction (PCR) amplification step. In PCR, double stranded DNA is “melted” apart at high temperature, then annealed to single stranded “primer” DNA at a lower temperature, that initiates extension of complementary sequence onto the original template DNA at an intermediate temperature. Copies of the original template DNA are then made for each thermal cycle. We will focus on temperature control for one of these steps, the actual the amplification process which can be accomplished at ~50 °C.

**Thermocouples/Peltier Cooling Effect**

Thermocyclers typically use Peltier cooling units to control the DNA sample temperature. The scientific principle behind the Peltier cooling technology is similar to that of a thermocouple. In a Peltier cooler, an electrical potential is applied across the junctions of two dissimilar metals, resulting in transfer of thermal energy from one junction to the other (i.e. one junction is heated while the other is cooled). Changing the polarity will reverse the direction of heat transfer, allowing one junction to be alternatively heated or cooled. In a typical Peltier unit, there are many small junction points and this arrangement is called a thermopile. Some complicated temperature measurement devices such as calorimeters also use thermopiles as a complex network of thermocouples to measure temperature around a sample in all three dimensions.

Peltier coolers are solid-state devices without any moving parts. The advantages include size, material cost, ability to cool below ambient temperatures, reliability, and low maintenance requirements. The disadvantage is that the cooling is not energy efficient and that heat from the hot side of the cooler must be constantly dissipated during operation. One of the main things to remember about a Peltier unit is that voltage only corresponds to a thermal energy *difference* between the two sides rather than a desired temperature at a particular side. Therefore if cooling is desired, then heat must be dissipated from the hot side so that the specified cold temperature is maintained. Alternatively, if heating is desired, heat must be directed to the cold side so that the specified hot temperature is maintained. While the rate of thermal energy transfer is proportional to the applied current between the junctions, application of current also results in ohmic heating throughout the device which is proportional to the square of current. Therefore there is a physical limit to how much a junction can be cooled even if the “hot” junction is maintained at ambient temperature.

**State 1**

Temperatures of each side are the same T_1_ = T_2_.

T_C_

**State 3**

Side 1 cooled. Heat pumped from cold side to hot side. Lowest T_C_ achieved by ΔT = T_H_ - T_C_. Must remove heat from hot side keep T_H_ from rising too high.

T_H_

T_H_

**State 2**

Side 1 Heated. Heat pumped from cold side to hot side. Highest T_H_ achieved by ΔT = T_H_ - T_C_. Must draw in heat to cold side keep T_C_ from falling too low.

T_C_

T_1_

T_2_

Figure 1. Illustration of temperature control for cooling and heating applications with a Peltier cooler.

The rate at which the Peltier unit can pump heat from its cold side to hot side is a function of the temperature difference between the two sides. The measured performance as supplied by the manufacturer Cui Inc. is given in Figure 2. At a given voltage applied to the terminals, e.g. 4 V, the top plot can be used to determine current draw. That current draw can then be located on the bottom plot to determine a relationship between the heat pumped and the temperature difference between the two sides.


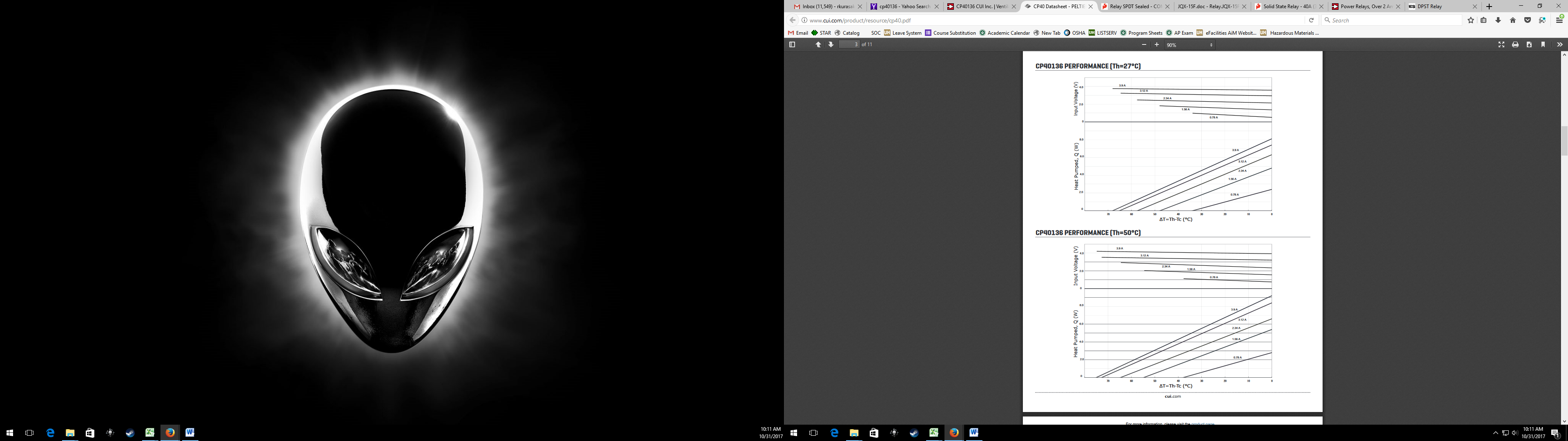


Figure 2. Performance of the CP40236 Peltier cooler at hot side temperatures of 27 and 50 °C.

**Methods**

The performance criteria that you will be evaluating are 1) rate of temperature change within the sample as well as 2) a uniform temperature distribution within the sample. You must design the heat sink such that you obtain a reasonable temperature ramping. You must also consider how to achieve a uniform temperature distribution within the sample.

Over the course of this multi-part laboratory exercise, there are several exercises to be completed. Remember, these are methods and the actual experimental objective(s) should be written in your own words.

- First generate an analytical model on this thermocycler system. These first calculations will serve as the basis for future decisions with the heat sink design and analysis of the results. Run models for both heating and cooling processes by changing the initial temperatures.
- Conduct simulations within Solidworks to further study not only the temperature ramping, but also the temperature distribution within the sample. Again, run models for both heating and cooling processes by changing the initial temperatures. Submit a final heat sink design for fabrication by the instructor.
- Design and conduct experiments on the thermocycler using the heat sink that the group has designed.
- Analysis will be made on all of the above results and design changes should be proposed if necessary.

Analytical Model

During the first period you will be performing basic energy balance analyses to characterize the temperature change over time for a sample block and heat sink fabricated from 6061 aluminum. The dimensions of the sample block and initial heat sink are 1” x 1” x 0.75” and 3” x 1” x 0.5” respectively. You may assume that heat conduction within the aluminum occurs much faster than all other processes such that the temperatures are uniform within the sample block and heat sink separately. Initially you may choose a constant power transfer between the two blocks, however you should refine your model to include a formula for the power transfer derived from the manufacturer’s data table for your Peltier unit. Assume that the sample block and heat sink exchange heat with the ambient air, but do not exchange heat with each other. This model will help determine the power transfer value to use in the computer simulation. You will need to look up an appropriate convective heat transfer coefficient for air. You may (should) use Matlab to execute the solution of this non homogeneous system and to plot your results.


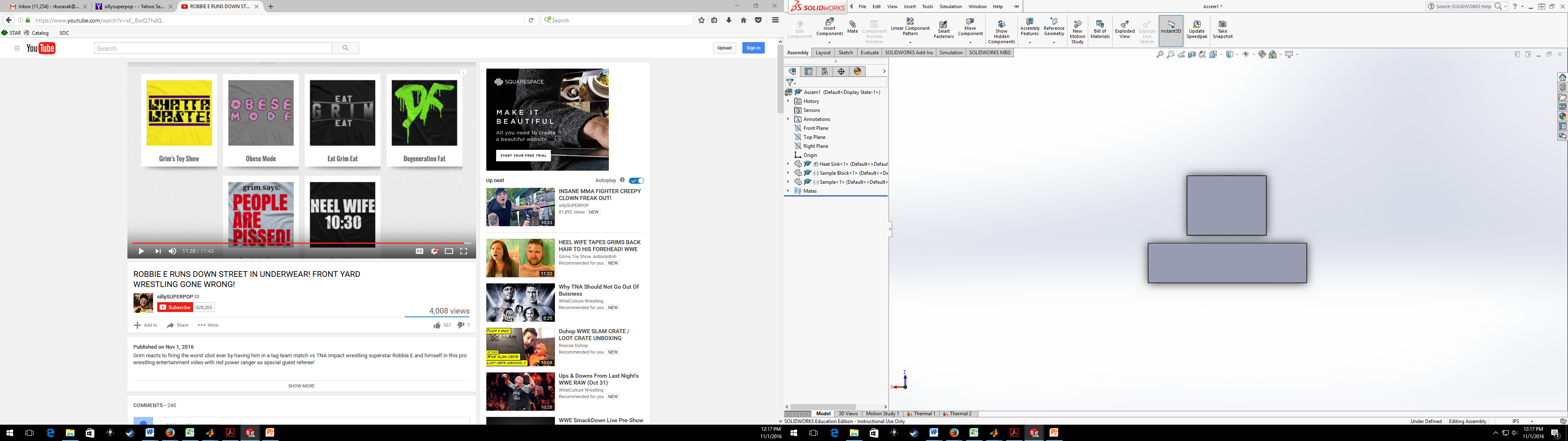


Figure 3. Layout of the sample block (above) and heat sink (below) in the thermocycler. Peltier unit not pictured.

*Heat Sink Design*

Alter the design of the heat sink either by changing the size or by adding fins or other geometries to enhance convective heat exchange. Design the heat sink to maintain temperatures that are desirable for the heating and cooling cycles of the thermocycler. Strike a balance between increasing the surface area while preserving the mass of the heat sink. The minimum and maximum dimensions for the heat sink are 1” x 1” x 0.5” and 1” x 3” x 0.5” respectively. Each participant should design at least one of their own heat sinks.

Solidworks Simulation

During the second period, you will be using Solidworks to create 3D drawings for the heat sink, sample block, and DNA (water) sample separately. Complete these drawing using the provided specifications. Solidworks will then be used to run multiple thermal simulations. Just as with your initial analyses of this system, you will need to define any process that will affect the energy stored in each component as well as the initial conditions, material properties, and boundaries. Solidworks will divide the parts into a finite number of elements and calculate the heat transferred between adjacent elements. This technique is called finite element analysis.

The simulation for the sample block and heat sink will be conducted separately. For the sample block simulation, insert the sample block and sample parts into an assembly and add the appropriate mating constraints to align the parts. This can be reviewed together in class.

1. Open a **New Study** under the **Study Advisor** drop down menu in the **Simulation** tab. Select a **Thermal** study for the current simulation. Take note of the parameters of the thermal study as seen under the **Feature Manager** window on the left.

- Parts – Select material composition of each part.
- Connections – Choose whether contacts between components will be insulated.
- Thermal loads – Processes that affect the energy stored in each component
  - Temperature – Can set an initial temperature of component or define a constant temperature to a surface.
  - Convection – Define a convective heat transfer process.
  - Heat Flux – Define a constant heat transfer flux process (power/area).
  - Heat Power – Define a constant heat transfer process.
  - Radiation – Define a radiative heat transfer process.
- Mesh – Create parameters to generate the finite element mesh.

1. Right click on the name of the study and choose to edit **Properties**. Change the study from a steady-state to a transient analysis and set the time range and time step.
2. **Add/Edit** the material for each part and select 6061-T6 aluminum for the sample block and heat sink. Select water for the sample.
3. Create an **Insulated** contact set.
4. Add a **Temperature** thermal load. Select **Initial Temperature** and select both components. Set the temperature to 25 °C. This thermal load must be the first one added.
5. Add a **Convection** thermal load. Look up an appropriate convective heat transfer coefficient and select an ambient temperature of 25 °C.
6. Add a **Heat Power** thermal load. Select the top face of the heat sink and define the heat power.
7. Add a **Heat Power** thermal load. Select the bottom face of the sample block and define the heat power.
8. Create a **Mesh** and then **Run** the simulation.
9. You can save a copy of the resulting surface plot as a jpeg file.
10. Create a **Section Clipping** to display the internal temperature uniformity of a slice.
11. Under **Plot Tools**, use the **Probe** option to select points along the cross-sectional slice and choose a **Response** plot to display how the temperature changes at specific points over time.


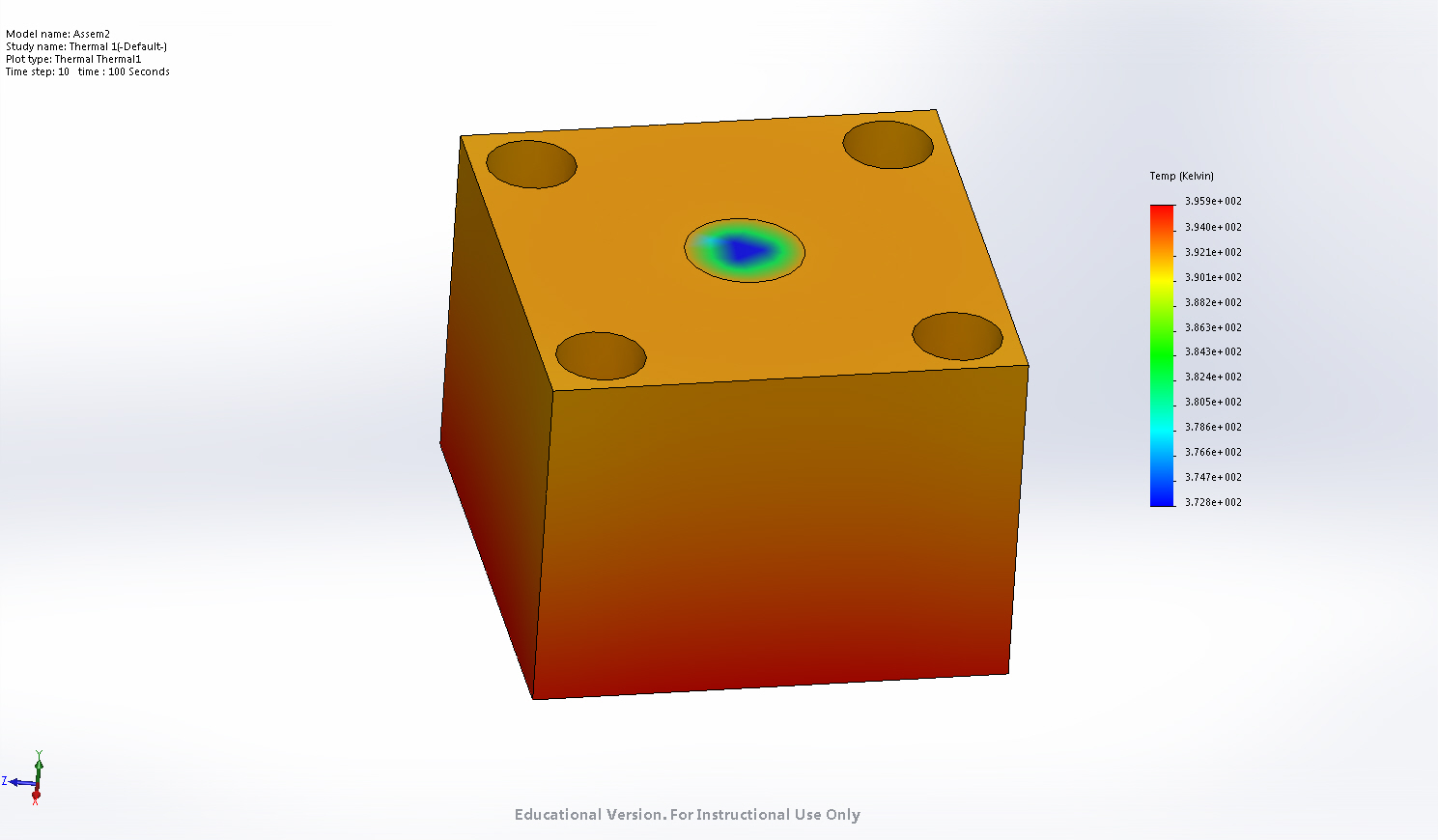


**Figure 4**. Color plot of the temperature distribution along the surface of an aluminum sample well block with heating along the bottom surface. Note that due to the relatively high thermal conductivity of aluminum the temperature distribution is mostly uniform.


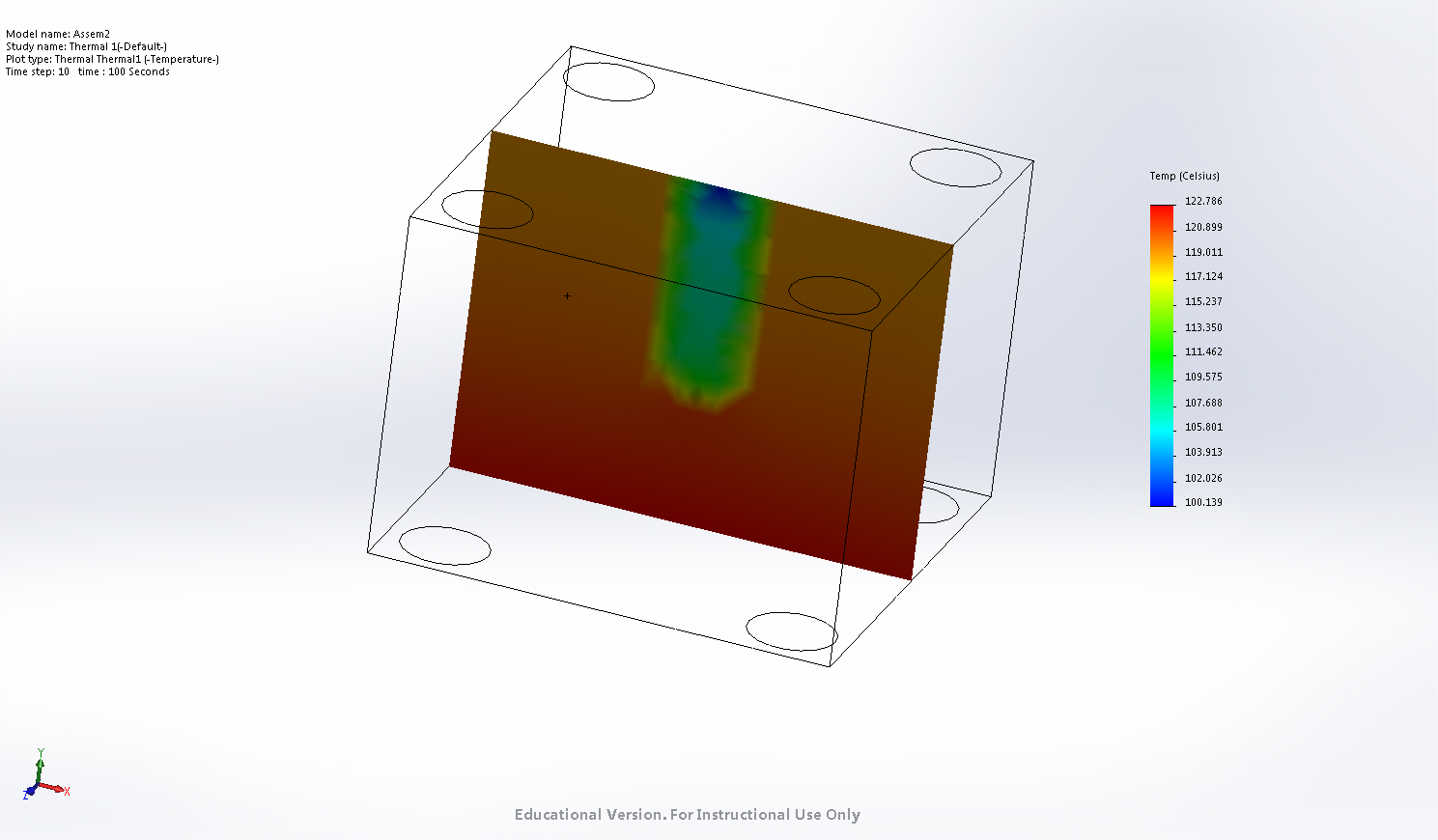


**Figure 5**. Temperature distribution of a cross section through the center of the middle reaction well. Notice that the bottom and sides of the well are hotter than along the top and center.


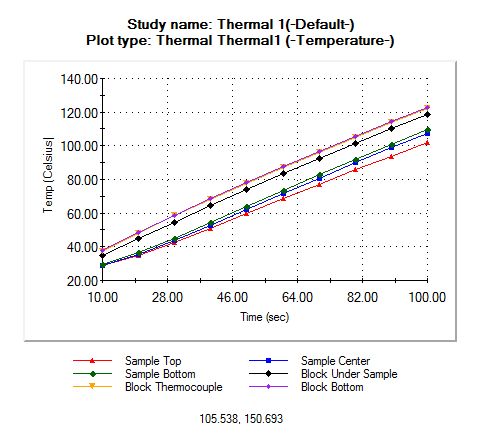


**Figure 6**. Transient temperatures simulated at multiple points along the sample well axis. Note that the bottom of the well responds faster and gets hotter than the top.

1. Right click on the thermal study tab and duplicate the study. You may choose to name the new study “cooling” or something similar. Modify the study to simulate cooling of the sample block and water sample assembly. Change the initial temperature to 50 °C and the reverse the direction of the heat power. Run the simulation again and save any desired results in tables or graphs.
2. Run cooling and heating simulations for the heat sink which you can conduct right from the part file. You do not need to create an assembly. Save any desired results. Each group member can run simulations with the heat sink they designed.
3. At this stage, you can also redesign your heat sink within the model window. Modify the heat sink by changing the length or cutting away material. Before you run new cooling and heating simulations, apply a mesh again. This will update the geometries in the thermal studies and you should not need to redefine all the parameters again.

Experimental Procedure

1. Design and conduct experiments to validate your simulation results. Make notes of any assumptions made during the simulation and design your experiment to be as consistent with most of these assumptions as possible. Each test that you run, may help you to not only assess the design but also refine your simulation settings, for example the convective heat transfer coefficient value or power addition/removal.

Minimum Report Requirements

1. The report should have the usual required sections.
2. Recall the two design criteria. There should be a timely temperature ramping to the desired temperature and the temperature of the sample should be relatively uniform.
3. There will be a lot of information that can go in the methods section and you will need to organize it and present it well. From the analytical model, you will get an idea for what the heat transfer power range of the Peltier unit will be as well as model for the temperature of the components as a function of time. This information along with your research into an appropriate convective heat transfer coefficient value will likely have a significant impact on the simulation results. How you determined these parameters should be explained in this section.
4. There can also be an abundance of graphs and results for your results and discussion section, so again organize and present the material well. Simulation and experimental results should be discussed here individually as especially as relating to the objectives as well as compared. After analyzing both results, you modified the simulation, discuss that here as well.
5. In the conclusion, briefly summarize the results in the context of the objectives. Also suggest design changes or the next step(s) in the design or development process.
