# Supplementary figures and images for: ABE-VIEW: Android Interface for Wireless Data Acquisition and Control
Source: Sensors (Basel). 2018 Aug 13;18(8):2647. doi: 10.3390/s18082647 (PMC6111993; doi:10.3390/s18082647)

Supplemental Material, Design S1 (Coffee Cherry Color Sorter)

Schematic


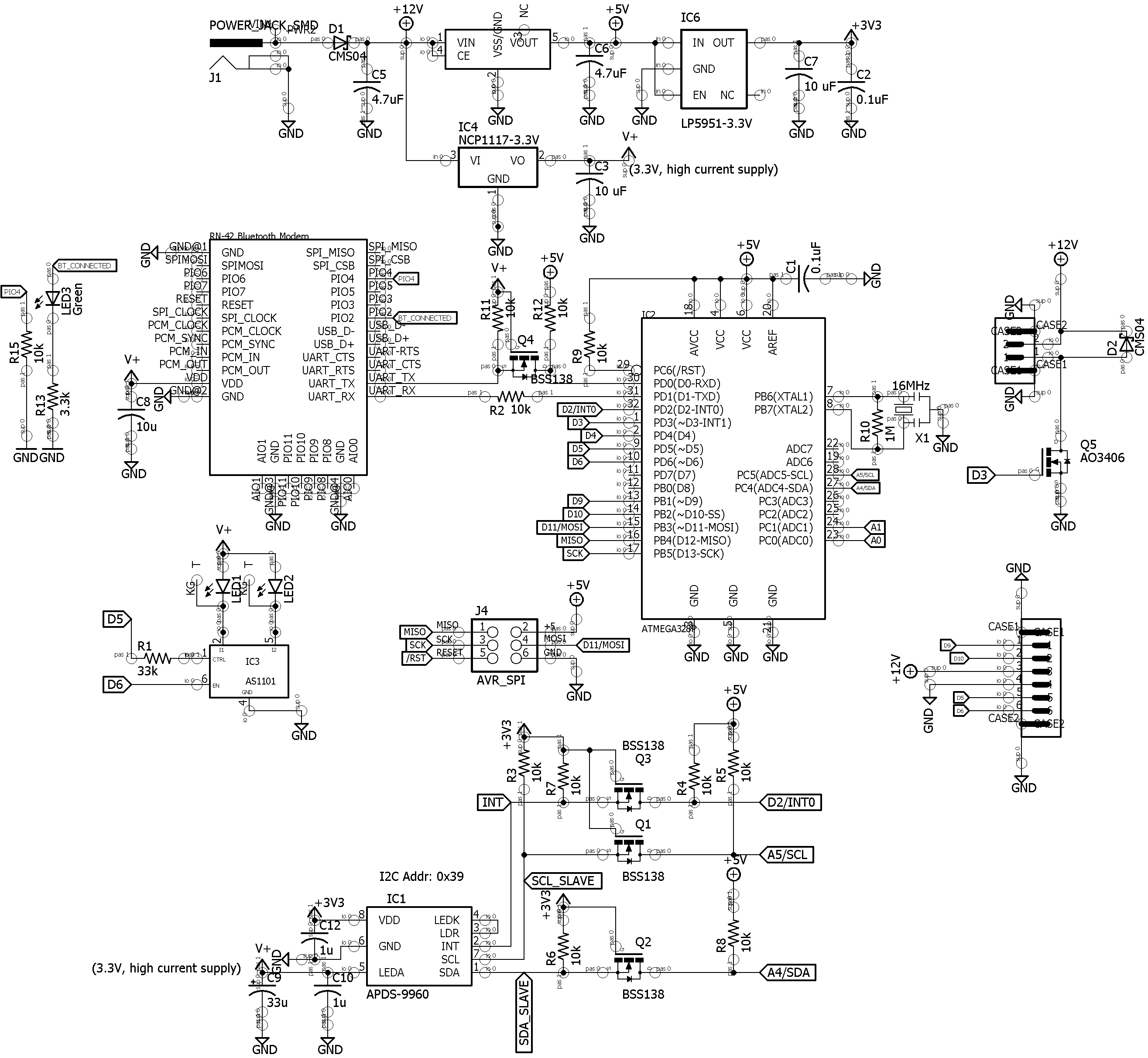


Layout


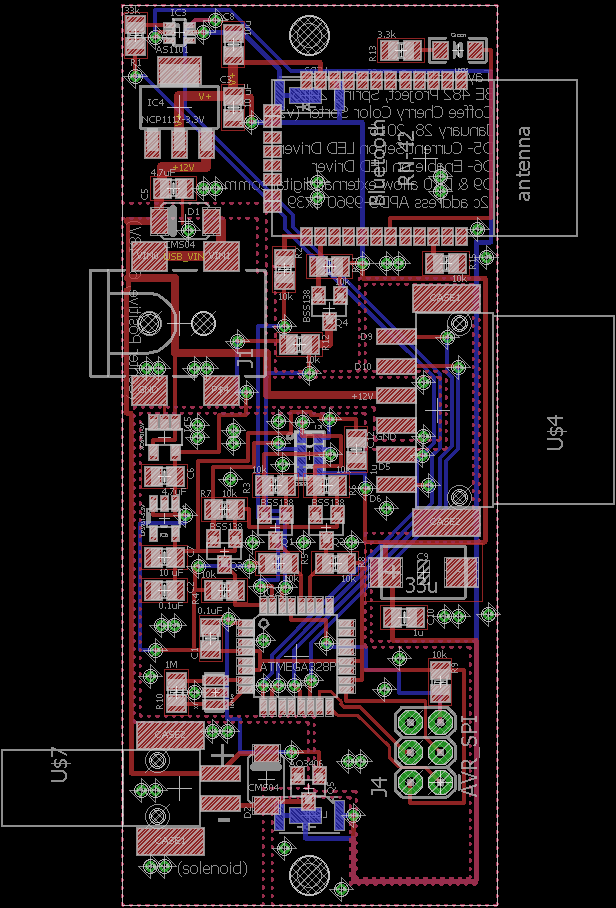

Supplement: Supplementary file 1 [file sensors-18-02647-s001.zip › Design S1/Design S1.docx]

Supplemental Material, Design S2 (ABE-Stat 1_0_01 Wireless Potentiostat Prototype)

Schematic


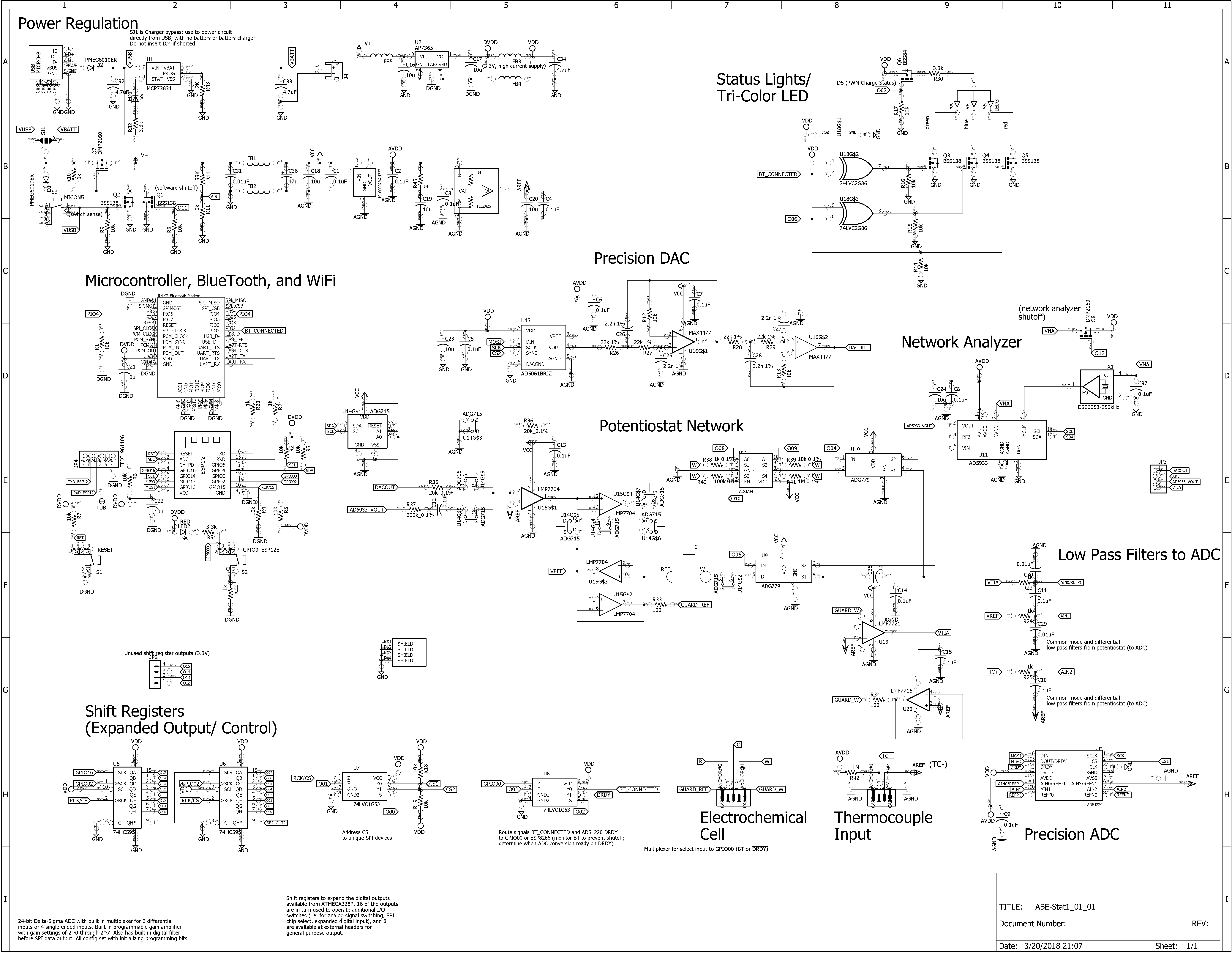


Layout


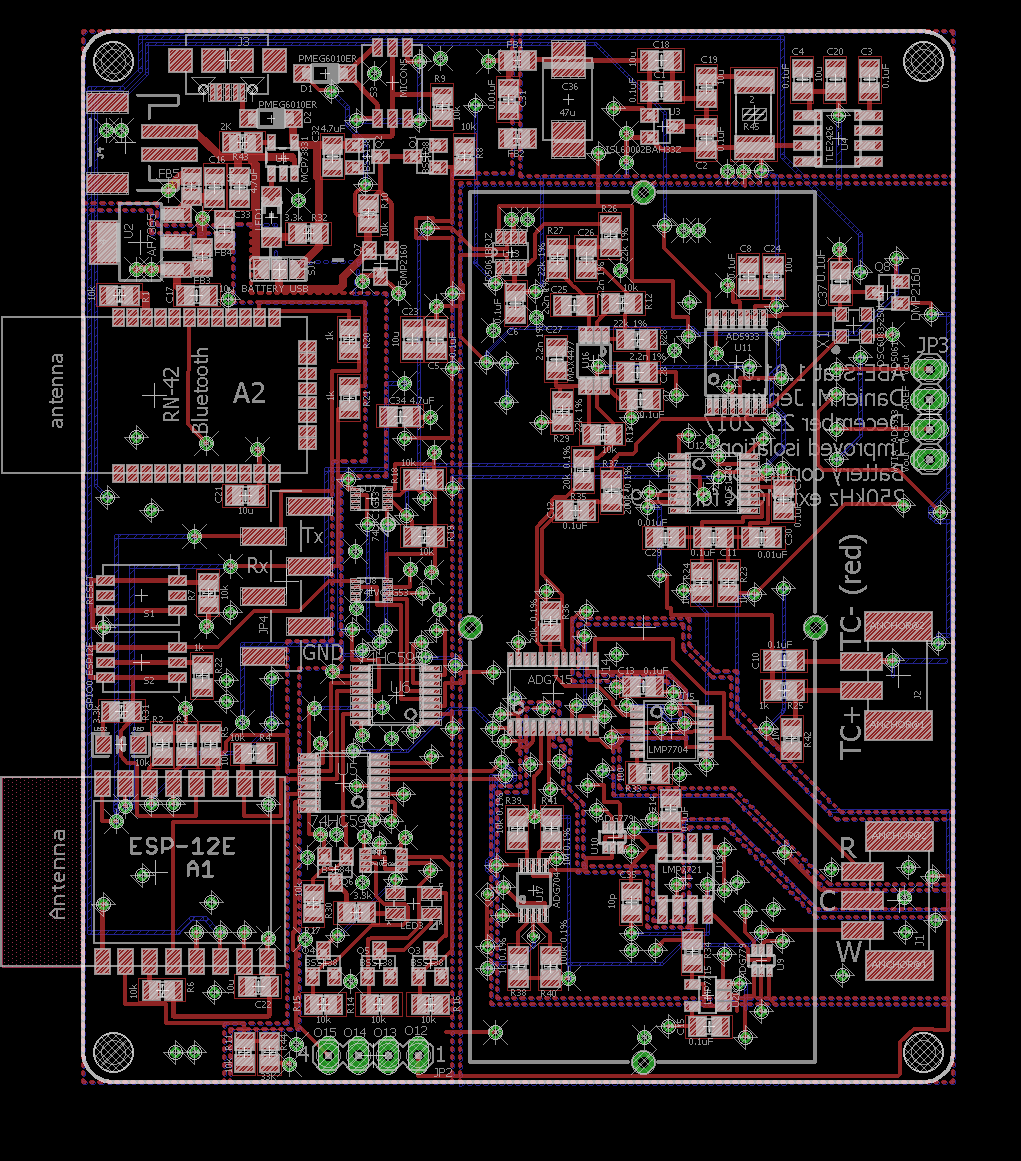

Supplement: Supplementary file 1 [file sensors-18-02647-s001.zip › Design S2/Design S2.docx]
